# Micro-Nano Bioactive Glass Particles Incorporated Porous Scaffold for Promoting Osteogenesis and Angiogenesis *in vitro*

**DOI:** 10.3389/fchem.2019.00186

**Published:** 2019-03-29

**Authors:** Ting Tian, Weihan Xie, Wendong Gao, Gang Wang, Lei Zeng, Guohou Miao, Bo Lei, Zhanyi Lin, Xiaofeng Chen

**Affiliations:** ^1^Guangzhou Higher Education Mega Center, School of Medicine, South China University of Technology, Guangzhou, China; ^2^Department of Biomedical Engineering, School of Materials Science and Engineering, South China University of Technology, Guangzhou, China; ^3^National Engineering Research Center for Tissue Restoration and Reconstruction, Guangzhou, China; ^4^Key Laboratory of Biomedical Materials and Engineering, South China University of Technology, Ministry of Education, Guangzhou, China; ^5^Key Laboratory of Oral Medicine, Guangzhou Institute of Oral Disease, Stomatology Hospital of Guangzhou Medical University, Guangzhou, China; ^6^Instrument Analysis Center, Frontier Institute of Science and Technology, Xi'an Jiaotong University, Xi'an, China; ^7^Department of Cardiology, Guangdong General Hospital, School of Medicine, South China University of Technology, Guangdong, China

**Keywords:** bioactive glass, micro-nano particles, nanocomposites scaffolds, bone regeneration, osteogenesis

## Abstract

Constructing the interconnected porous biomaterials scaffolds with osteogenesis and angiogenesis capacity is extremely important for efficient bone tissue engineering. Herein, we fabricated a bioactive micro-nano composite scaffolds with excellent *in vitro* osteogenesis and angiogenesis capacity, based on poly (lactic-co-glycolic acid) (PLGA) incorporated with micro-nano bioactive glass (MNBG). The results showed that the addition of MNBG enlarged the pore size, increased the compressive modulus (4 times improvement), enhanced the physiological stability and apatite-forming ability of porous PLGA scaffolds. The *in vitro* studies indicated that the PLGA-MNBG porous scaffold could enhance the mouse bone mesenchymal stem cells (mBMSCs) attachment, proliferation, and promote the expression of osteogenesis marker (ALP). Additionally, PLGA-MNBG could also support the attachment and proliferation of human umbilical vein endothelial cells (HUVECs), and significantly enhanced the expression of angiogenesis marker (CD31) of HUVECs. The as-prepared bioactive PLGA-MNBG nanocomposites scaffolds with good osteogenesis and angiogenesis probably have a promising application for bone tissue regeneration.

## Introduction

Bone defects, caused by breaks, tumors, and traumas, bring great pressure to public health, and become an urgent problem to be resolved (Li et al., 2018a; Zheng et al., 2018). In recent years, bone tissue engineering scaffolds play a more and more important role in bone repair field due to the growing market demand (Xu et al., 2016; Zhao et al., 2018a), due to the drawbacks in autologous and allograft bone graft (Zhang et al., 2017). Bone scaffolds should have the ability to mobilize cells to reach the lesion place after implanted, until the regenerated tissue is enough stabilized to support the native bone. Thus, the scaffolds need to serve as extracellular matrices (ECMs) temporarily to provided structural support and facilitate cells survival, attachment, proliferation, and differentiation, with the final objectives of generating functional bone tissue (Rath et al., 2012; Do et al., 2015; Raeisdasteh et al., 2017; Martins et al., 2018). Unfortunately, the bone repair scaffolds still have many problems, such as the lack of biological activity of bonding with nature bone and angiogenetic ability (Li et al., 2018b). In fact, based on the bionics principle and the previous reports about complex architecture of bone tissue, three-dimensional (3D) scaffolds with porous structure and functional inorganic particle are considered to be one of a promising means to resolve above problems and achieve the purpose of bone tissue repair (Lei et al., 2012; Chen and Liu, 2016; Chen et al., 2016).

As we all know, the bone matrix is composed of inorganic substance (mainly nanoscale hydroxyapatite) and organic substance (mainly type I collagen) (Yi et al., 2018). Especially, the bioactive nanocomposites biomaterials mimicking the structure of native tissues have shown promising application in tissue regeneration (Xi et al., 2018; Li et al., 2019; Wang et al., 2019). To mimic the composition of bone, constructing composite scaffolds with organic and inorganic component will be meaningful for bone repair (Lei et al., 2018; Martins et al., 2018). Among the biodegradable polymers, PLGA is a suitable synthetic biomaterial and it is widely used in tissue engineering field owing to its biocompatibility, tailored biodegradation rate (depending on the molecular weight and copolymer ratio) to match those of the tissues. Meanwhile, it can construct high porosity and interconnected pores of 3D structure, which is crucial for ideal scaffolds and cell behaviors (Gentile et al., 2014; Lei et al., 2018).

However, the pure PLGA scaffold has a low mechanical property, poor bone-bonding bioactivity and osteogenic activity (Bose et al., 2012; Zhou et al., 2014; Li et al., 2016). To solve the problem above, many functional inorganic particles such as silica, beta-TCP and hydroxyapatite have been incorporated into PLGA, which dramatically improved the mechanical property and osteogenesis performance (Boccaccini et al., 2010). However, the inorganic nanoparticles-incorporated PLGA scaffolds still show low bone-bonding activity and angiogenesis ability. Micro-nano bioactive glass (MNBG) as a kind of bioactive biodegradable biomaterial (Hu et al., 2013; Xue et al., 2019), has a good ability of mineralization forming into hydroxyapatite and excellent biological activity (Rahaman et al., 2011; Bachar et al., 2016). Furthermore, the ions (Si^4+^ and Ca^2+^) released from MNBG have therapeutic functions, which can stimulate cell proliferation and the expression of osteogenesis and angiogenesis related genes, eventually to facilitate osseointegration (Xynos et al., 2001; Gorustovich et al., 2010; Gerhardt et al., 2011; Hoppe et al., 2011; Cong et al., 2015; Zhang et al., 2016; Dashnyam et al., 2017; Mao et al., 2017; Kargozar et al., 2018). As a result, PLGA compound with MNBG (to be 3D scaffold) not only complement each other but facilitated bone repair performance as well. In fact, PLGA/BG scaffolds have been prepared in previous studies (Zhou et al., 2014; Cui et al., 2017; Kim et al., 2017). However, the composite scaffold they prepared with an irregular and sharp pore structure which was not conducive to cell growth and migration. The poor bonding between organic and inorganic was also affected the ion release and mechanical properties of the scaffold, in the end, resulted to the poor bone repair.

Herein, we aim to employ the phase separation method to fabricate porous PLGA-MNBG nanocomposite scaffold and investigate their physiochemical/biological properties. The morphology, physicochemical properties and *in vitro* biocompatibility of the PLGA-MNBG composite scaffolds were investigated in detail (Qian et al., 2014). We hypothesize that the addition of MNBG might be effective in enhancing the mechanical properties, osteogenic activity and angiogenesis of PLGA scaffolds *in vitro*.

## Materials and Methods

### Materials

Dodecyl amine (DDA, sigma) was used as the mesopore template, calcium nitrate tetrahydrate (CN; Ca(NO_3_)_2_·4H_2_O), absolute ethanol (anhydrous, 99.8%, C_2_H_5_OH), tetraethyl orthosilicate (TEOS; Si(OCH_3_)_4_), and triethylphosphate (TEP) were purchased from Guangzhou to fabricate the micro-nano bioactive glass. Poly (lactic-co-glycolic) acid (PLGA, lactic: glycolic molar ratio = 50:50, Mn: ~8.8 × 10^4^ g/mol, Jinan, Shandong) was used as a matrix for 3D scaffold. 1, 4-Dioxane (C_4_H_8_O_2_, Tianjin) were used as solvent and pore forming materials.

### Preparation of Micro-Nano Bioactive Glass Particles

MNBG (60% SiO_2_, 36% CaO, and 4% P_2_O_5_) were produced by sol-gel method combined with DDA as template. In brief, DDA was first dissolved in deionized water and absolute ethanol with magnetic stirring at 40°C. Then TEOS, TEP, and CN solution were sequentially added to the above solution drop-wise by injector pump (0.5 mL/min) in proportion. After 3 h, stop stirring and let the resultant solution stand overnight for the precipitation of the white gel. The white precipitate was collected, rinsed and then freeze-drying for 48 h. Finally, the dried gel was sintered at 650°C for 3 h to obtain the MNBG particles.

### Preparation of Scaffolds

Pure PLGA scaffold (0% MNBG) and different composition of PLGA-MNBG composite scaffolds (10, 20, 30, and 40% MNBG) with porous structure were prepared. In brief, 1 g of PLGA was dissolved in 10 mL of 1, 4-dioxane to produce control group (0% MNBG) with porous structure. For the PLGA-MNBG composite scaffolds with different composition, different amount of MNBG [10, 20, 30 and 40% w/w_s_ (w_s_: solid content was constant as 10% w/v)] were well dispersed within the solvent and then followed by the addition of PLGA. The mixture slurry was poured into a polytetrafluoroethylene (PTFE) mold, and then frozen at −20°C overnight and lyophilized for 3 days.

### Properties of PLGA-MNBG Composite Scaffold

The morphology of MNBG was investigated by field emission scanning electron microscope (SEM, Merlin Carl Zeiss Jena). The X-ray diffraction analyzer (XRD, Bruker D8, Netherlands) at a scanning speed of 2°/min and 2θ from 10 to 70° was used to detect the phase composition of MNBG. The microstructure of obtained scaffolds was observed by SEM (FEI Quanta 25, USA) with energy dispersive spectroscopy (EDS). Fourier transform infrared spectroscopy (FTIR; Vector 33, Bruker, GER) were used to detect the chemical bond of the scaffold in the range of 400–2,000 cm^−1^ with 4 cm^−1^ resolution averaging 50 scans. The compressive mechanical properties of the scaffolds were performed by uniaxial compression tests using a universal testing machine (Instron 5967, USA) and the experiments were performed at room temperature with a cross-head speed of 1 mm/min and a load cell of 5.0 kN. Five testing samples for each group and were made into cylinders with a diameter of 8 mm and a height of 9 mm.

The degradation property of composite scaffolds were detected by immersing 30 mg samples in 30 ml of simulated body fluid (SBF) with an initial pH of 7.4 at 37°C, and stirred at 100 rpm. At every time points (3, 7, 14, 21, 28 d), collected the scaffolds and rinsed with water, then dried prior to weigh. The remaining weight was expressed using the following equation:

$$\begin{array}{l} \text{Remaining weight}\left(\text{\%}\right)={\text{W}}_{\text{d}}/{\text{W}}_{\text{o}}\times \;100\% \end{array}$$

W_d_ is the weight after degradation. W_o_ is the original weight of the scaffold.

The Apatite formation property of composite scaffolds were detected by immersing 30 mg samples in 30 ml of simulated body fluid (SBF) with an initial pH of 7.4 at 37°C, and stirred at 100 rpm. After 3 and 7 days incubation, collected the scaffolds and dried prior to detect the phase composition by X-ray diffraction analyzer (XRD, Bruker D8, Netherlands) at a scanning speed of 2°/min and 2θ from 10 to 70°.

### *In vitro* Cellular Evaluation of Composite Scaffold

#### Cell Culture

Mouse bone mesenchymal stem cells (mBMSCs) were cultured in DMEM with 10% fetal bovine serum and 1% penicillin-streptomycin (P/S) and were used to investigate the cell behaviors on the porous scaffold. Human umbilical vein endothelial cells (HUVECs, purchased from ScienCell, USA) was used to analysis angiogenesis performance of cells on the scaffold. HUVECs were cultured in endothelial cell medium (ECM, ScienCell, USA) with 5% FBS, 1% penicillin-streptomycin (P/S) and 1% endothelial cell growth supplement/heparin kit (ECGS/H, Promocell). The scaffold (2 mm height and 8 mm diameter) were placed into the 48-well plates, sterilized by immersing in 75% ethanol overnight and washed with PBS for three times by 30 min interval. 4 × 10^4^ mBMSCs were seeded onto each scaffold and 5 × 10^4^ HUVECs were seeded onto each scaffold. All plates were placed in the 37°C humidified with 5% CO_2_ incubator and refreshed culture medium every 2 days until harvest.

#### Cell Attachment

For cell attachment testing, the scaffolds were harvested at 3 days and washed with phosphate-buffered saline (PBS) for twice, fixed with 2.5% glutaraldehyde at 4°C for 4 h. Then the scaffolds were immersed into gradient ethanol (30, 50, 70, 80, 90, 95, and 100% v/v) for 10 min dehydration in sequence, dried in air and observed by SEM (FEI Quanta 25, USA).

#### Cell Viability and Proliferation

The cell viability cultured on composite scaffold at day 1 and 5 was evaluated using a calcein-AM/PI double stain kit (Bio Vision, USA). The cell-seeded scaffolds were rinsed with PBS and added with the mixture solution of 1 μM calcein-AM and 3 μM PI. After 30 min incubation, the morphology of stained cells was observed by a confocal laser scanning microscopy (CLSM, Leica SP8, Germany).

The Cell Counting kit-8 (CCK-8, Dojindo, Japan) was used to evaluate the proliferation of mBMSCs on the scaffolds. In brief, the scaffolds were harvested at 1, 4, 7 days and transferred to a new 48-plates, then working solution (1:9 ratio of CCK-8 solution: medium) was add to each well and incubated at 37°C, 5% CO_2_ for 1 h in dark. The optical density (OD) value was detected at 450 nm using a micro-plate reader (Thermo 3001, USA). Five samples for each group.

#### Alkaline Phosphate (ALP) Activity

The ALP activity was cell differentiation marker of osteoblastic at the early stage. In order to evaluate the early osteogenic differentiation ability of mBMSCs seeded on the scaffolds, the ALP expression was quantified at 7 and 14 days by Alkaline Phosphatase Assay Kit (Beyotime, China). That is, the harvested scaffolds were transferred to a new 48-well plate, washed with PBS three times and added RIPA Lysis Buffer (Beyotime, China) to extract ALP. After centrifugation and collecting supernatant, 20 μL samples, 30 μL buffer solution and 50 μL chromogenic substrate was added to a 96-well plate in sequence, mixed and 100 μL stop buffer were used to stop the reaction, then measured the ALP activity at 405 nm using a micro-plate reader. Five scaffolds for each test group.

#### Angiogenesis of Endothelial Cells

The angiogenesis effect was evaluated by cell viability and immunofluorescent staining of CD31 of HUVECs. The cell viability was detected as the mBMSCs at day 1, 4, and 7. Immunofluorescent staining of CD31 was evaluated after 3 days culturing. In brief, the cells on scaffold were first fixed 30 min with 4% paraformaldehyde and washed with PBS. Then permeabilized 10 min with 0.1% Triton X-100 and washed with PBS. After that, blocking with bovine serum albumin (BSA) for 1 h and using primary antibody of CD31 (1:100 dilution; abcam, USA) to incubate at 4°C overnight. After washing with PBS, goat anti-mouse immunoglobulin G H&L (1:1000 dilution; abcam, USA) was used to incubate for 1 h. At last, the nuclei of HUVECs were stained with DAPI (Beyotime). The stained cells were observed by a confocal laser scanning microscopy (CLSM, Leica SP8, Germany).

### Statistical Analysis

All experiments were performed in triplicate. Data were expressed as mean ± standard deviation (SD). A one-way analysis of variance (ANOVA) was used to analyse differences between the experimental groups. The statistically significant was considered at the ^*^p < 0.05, ^**^p < 0.01, or ^***^p < 0.001. Statistical analysis was performed using Graph Pad Prism 5 software.

## Results and Discussion

### Characterizations of the MNBG Particles and PLGA-MNBG Scaffolds

**Figure 1:**
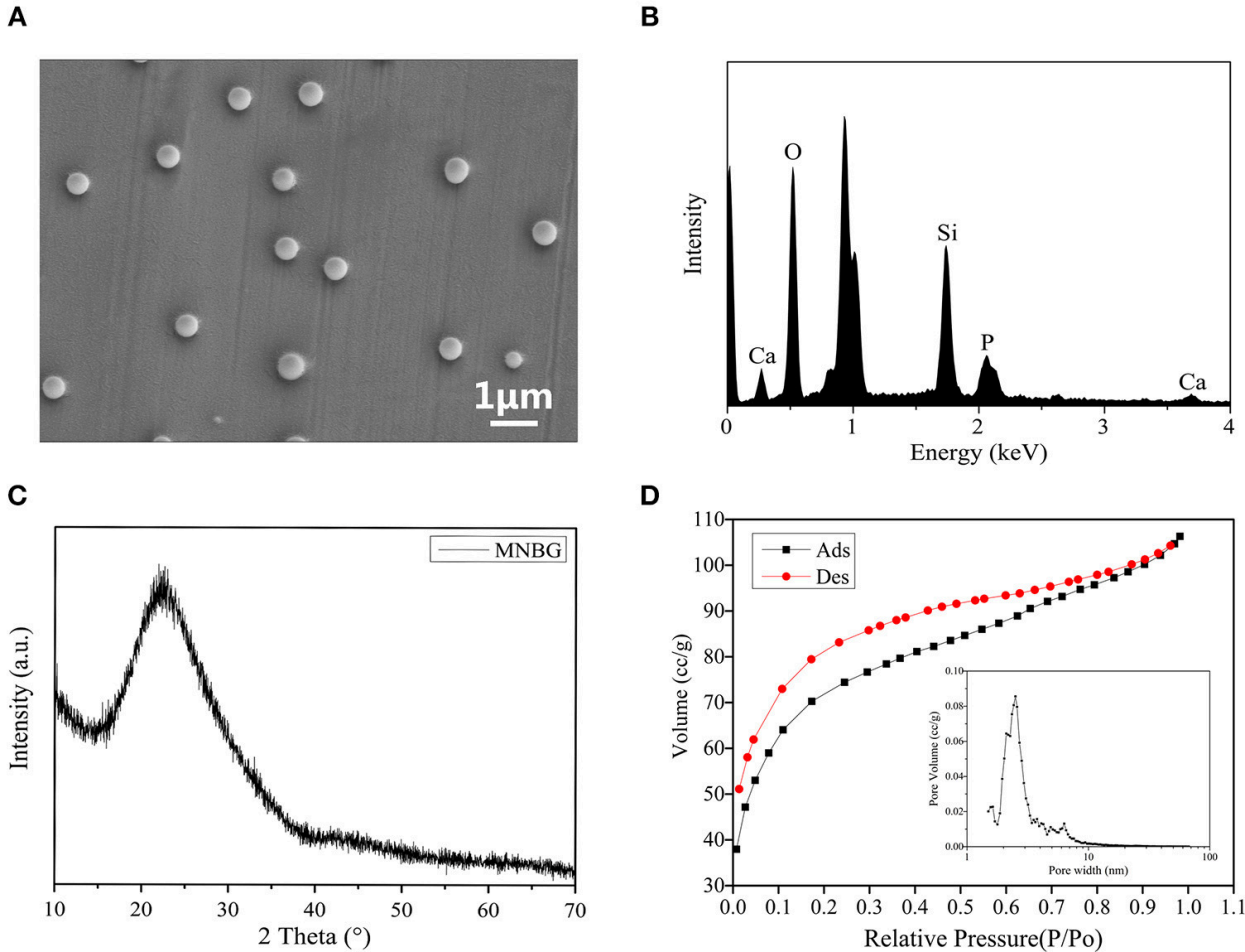
Characterizations of MNBG. **(A)** SEM image indicating the morphology of MNBG with uniform monodisperse sphere. **(B)** EDS spectra showing the existence of Si, Ca and P. **(C)** XRD pattern demonstrating the representative amorphous state of MNBG. **(D)** The N_2_ adsorption-desorption isotherms and pore size distributions of MNBG.

The monodisperse MNBG spheres were successfully prepared by a sol-gel method combined with DDA as template and detected by the SEM image (Figure 1A) with an average diameter about 440 nm (Figure S1). The EDS graph showed the existence of Si, Ca and P in MNBG (Figure 1B). The XRD analysis showed a diffuse peak at ~23° (2θ), which was the characteristic of glassy state, indicating the representative amorphous structure of MNBG (Figure 1C) which could contribute to the ion release (Figure S2). From the N_2_ adsorption-desorption isotherm curve (Figure 1D), it was seen that the curve was consistent with the type IV adsorption-desorption curve, and the hysteresis loop was type H3 which was the characteristics of mesoporous structure. The pore size distribution diagram indicated that most of the pores in MNBG were between 2 and 10 nm and the average pore size was 2.5 nm, and the smaller mesoporous structure was probably the slit pores formed by the continuous accumulation of nano particles.

**Figure 2:**
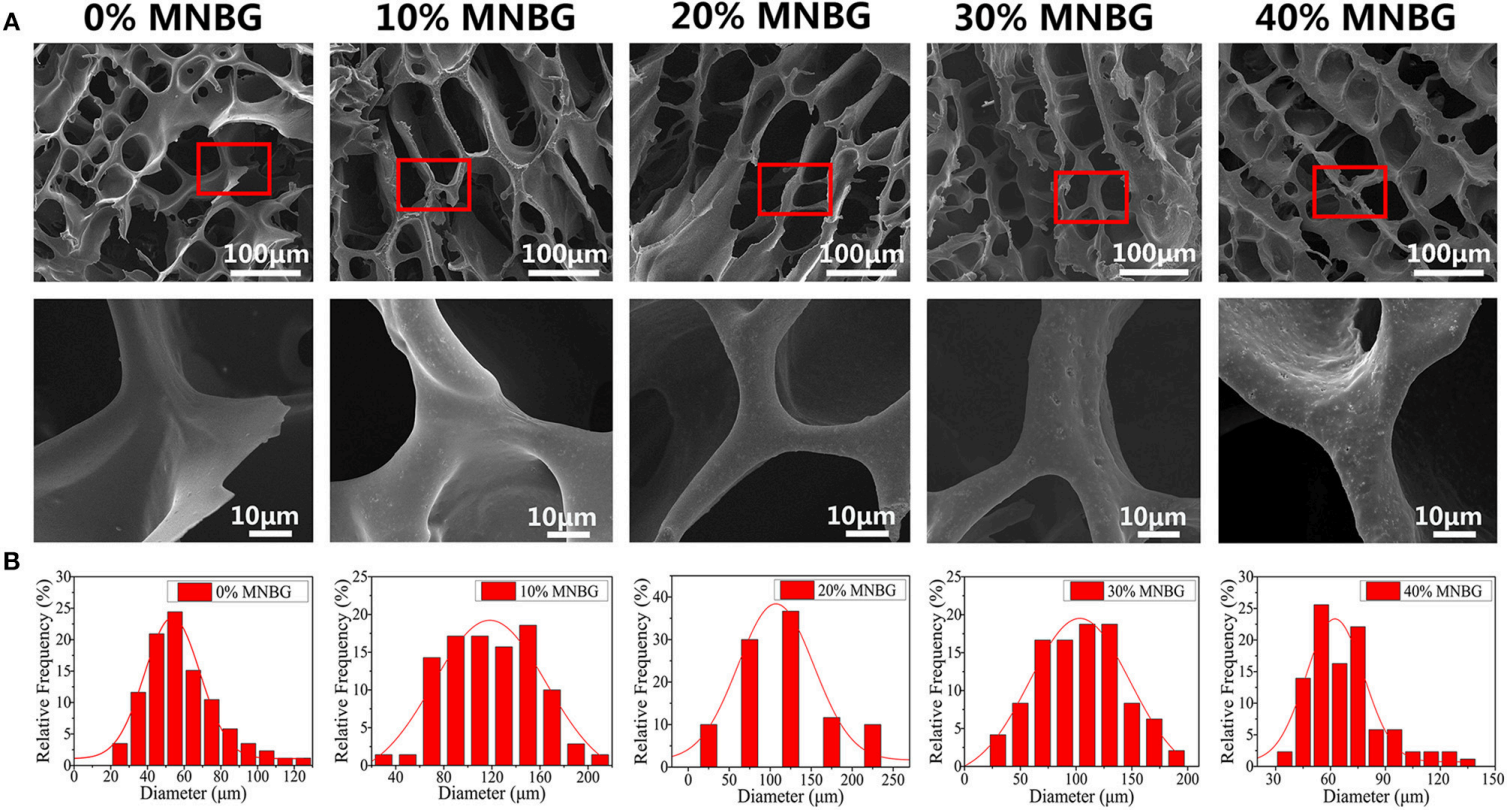
Characterizations of PLGA-MNBG scaffold. **(A)** SEM image showing the porous structure of composite scaffolds. **(B)** Pores diameter distribution of different composition of PLGA-MNBG scaffolds.

The composite scaffolds (0, 10, 20, 30, 40% MNBG, w/w) were prepared by freeze-drying technique, the structure and pore size of composite scaffolds were detected by SEM. The image (Figure 2A) showed that the scaffolds possessed highly interconnected porous structure, MNBG was well dispersed in the PLGA matrix and the incorporated MNBG could enlarge the pore size obviously (Figure 2B). The average pore size of composite scaffolds of 0% MNBG group was 58.0 ± 15.64 μm, 10, 20, 30, 40% MNBG groups was 120.27 ± 35.58, 120.60 ± 45.40, 109.77 ± 33.51, and 68.74 ± 19.68 μm, respectively (Figure 2B), indicating the significant increase of pore size at 10% and 20% MNBG (100–300 μm). The improved pore size was probably due to that the MNBG could weaken the intertwined PLGA chains and limited the movement of polymer chains to a certain extent to make it larger ice crystal and resulted larger pore size. Whereas, when the MNBG incorporation was up to 40% w/w, the effect of PLGA wrapping on MNBG makes the wall of the scaffold thickened, thus resulting in the reduction of the pore diameter of the scaffold. In fact, the increased pores size was more helpful to the survival and metabolism of cell (Laschke et al., 2006; Wu et al., 2013). In addition, the FTIR graph (Figure S3) of MNBG and PLGA-MNBG composite scaffolds showed the infrared absorption band is the typical asymmetric stretching vibration peak of Si-O-Si in bioactive glass, the symmetric stretching vibration peak of Si-O and the curved vibration peak of Si-O-Si at 1,090, 800, and 475 cm^−1^ in the graph of MNBG (Li et al., 2018). While the graph of PLGA-MNBG at 3,000, 1,456, 1,759, and 1,200 cm^−1^ showed the saturated hydrocarbon bonds, ester carbonyl and ester ether bond of PLGA. Meanwhile, the EDS mapping of PLGA-MNBG scaffolds (Figure S4) detected the distribution of Si from MNBG. The results showed that the Si in 0% MNBG group (without MNBG) was not detected and the MNBG-incorporated groups appeared uniformly Si element signals, suggesting the uniform distribution of MNBG in the PLGA-MNBG scaffolds.

### Mechanical Property of PLGA-MNBG Composite Scaffolds

**Figure 3:**
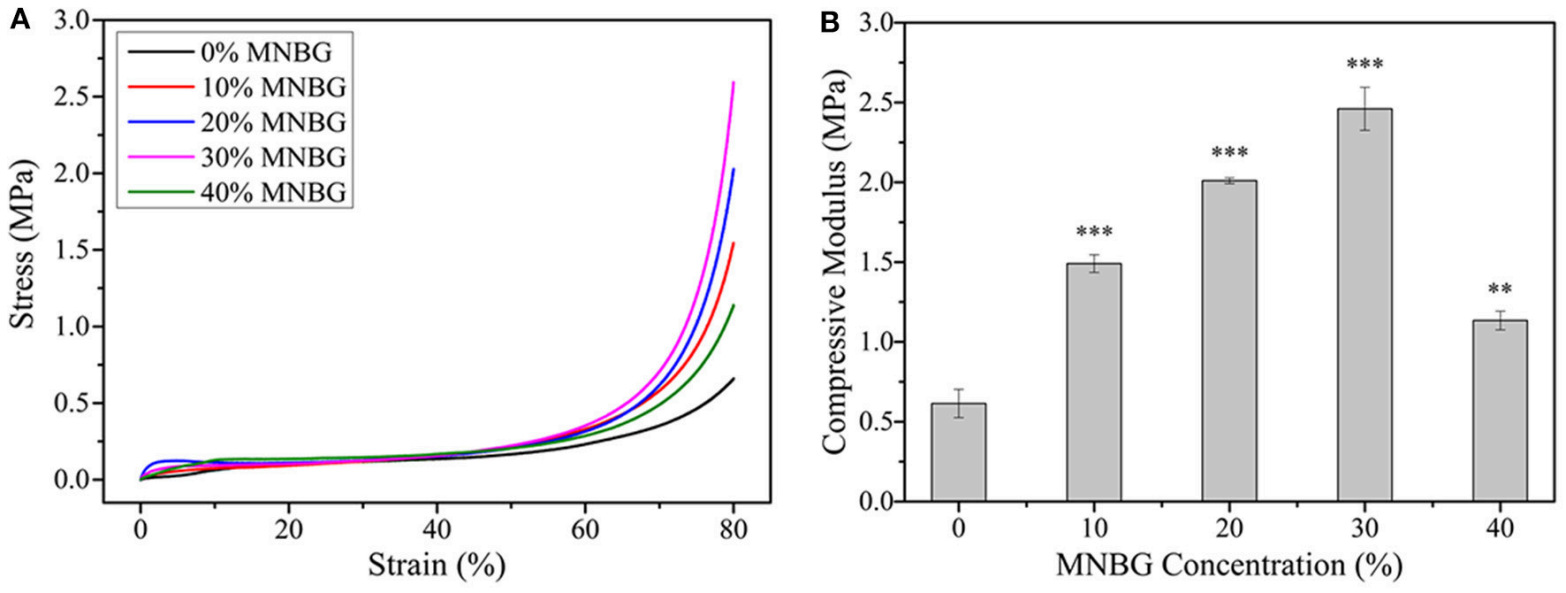
Mechanical property evaluation of PLGA-MNBG composite scaffolds. **(A)** Compressive stress-strain curve as various content of MNBG. **(B)** Compressive modulus of composite scaffolds depended on MNBG concentration (^*^p < 0.05, ^**^p < 0.01, ^***^p < 0.001 compare to 0% MNBG).

The mechanical performance of composite scaffolds were evaluated by uniaxial compression testing, The results (Figures 3A,B) showed that the stress increased with the incorporated amount of MNBG, the compressive modulus of scaffolds increased as well until the MNBG incorporation was up to 40%w/w. The compressive modulus of 0% MNBG group was 0.61 ± 0.09 MPa, 10, 20, 30, and 40% MNBG groups were 1.49 ± 0.05, 2.01 ± 0.02, 2.46 ± 0.13 and 1.13 ± 0.058 MPa, respectively. The compressive modulus of scaffolds with MNBG groups were approximately 2.4-, 3.3-, 4.0-, and 1.85- folds, respectively, >0% MNBG group. The improvement of the compressive modulus of composite scaffolds was resulted from the existence of bioactive glass particles which inhibit the movement of PLGA molecules chain. At the same time, the improvement of the compressive modulus were meaningful for scaffold to match well with nature bone after implanting (Raeisdasteh et al., 2017).

### Biodegradation Property and Apatite-Forming Activity of PLGA-MNBG Scaffolds

**Figure 4:**
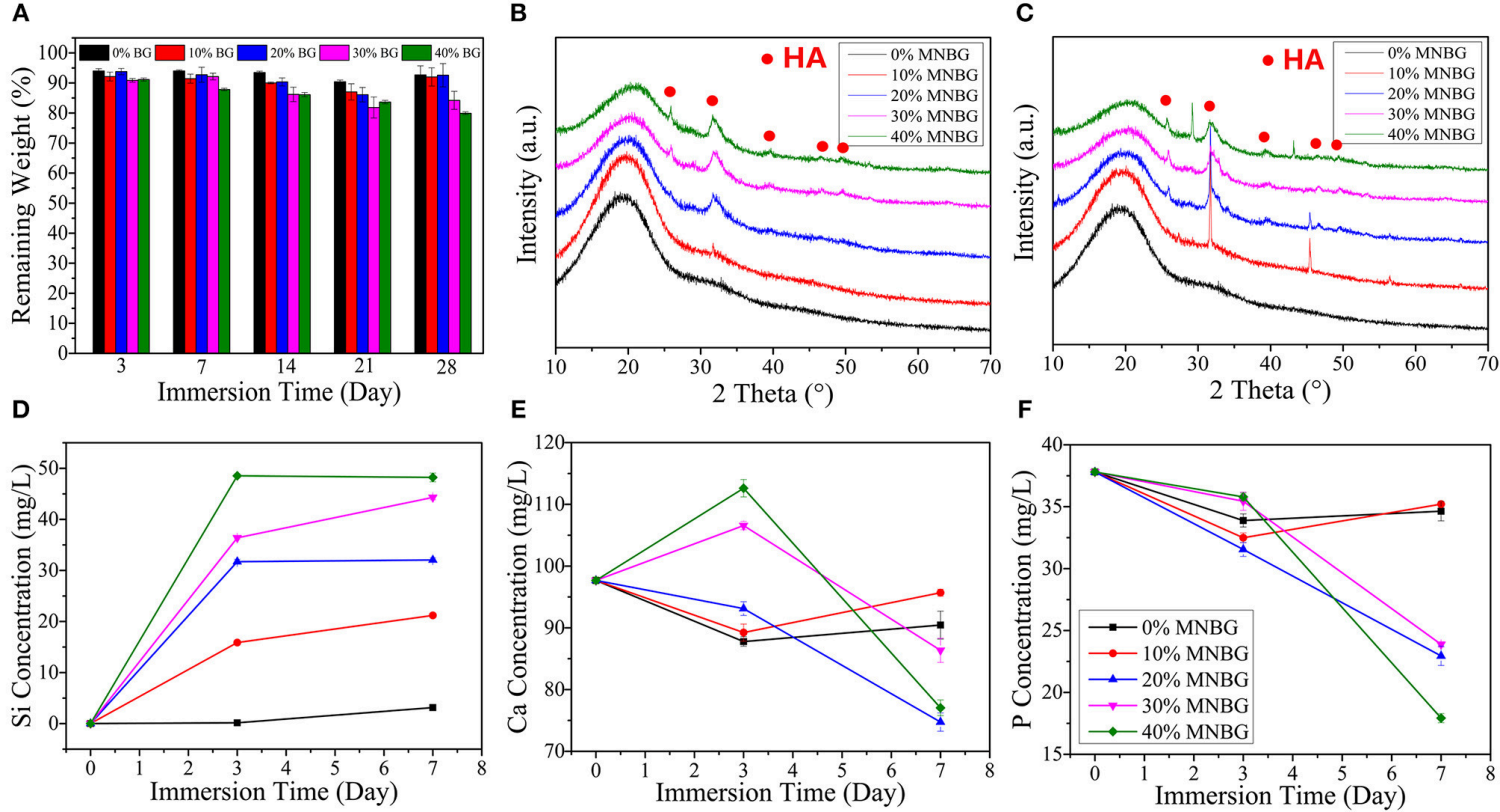
Biodegradation and apatite-forming ability of composite scaffolds in SBF. **(A)** Mass loss behaviors of scaffolds in SBF during 28 days immersing. **(B,C)** XRD patterns of scaffolds after soaking in SBF for 3 days **(B)** and 7 days **(C)**. **(D–F)** Ions release curves of scaffolds for **(D)** Si; **(E)** Ca; **(F)** P after soaking in SBF for 3 and 7 days.

The physiological stability of composite scaffolds is essential for bone repair after implanting, as degradation rate should match with the bone in-growth rate and improve subsequent remodeling to achieve the functional recovery (Levengood and Zhang, 2014). The mass loss measurement in SBF showed that all groups of scaffolds were well-resistance to hydrolysis until 28 days (Figure 4A). At day 3, the weight of scaffolds had a little big drop because of the und MNBG particles which had run off the scaffold surface. As prolonged the immersion time, 0, 10, and 20% MNBG groups had slight weight change and even had a observably weight increase after 28 days immersing, while with the increase of MNBG (30% MNBG, 40% MNBG groups), it was continuous declination though the remaining weight (%) is still higher than 80%. This effect could be explained by the balance between degradation and apatite-forming (Guo et al., 2017). When the amount of MNBG was < 30% w/w, it could be integrated with PLGA firmly and the apatite formed simultaneous with MNBG and PLGA degradation would be inhibited. Instead, when higher than 30% (w/w), MNBG loss greatly and could not form apatite masking with resulted continuous decrease of weight (Varila et al., 2012).

Hydroxyapatite (HA) is generated on the surface of bioactive glass when it contact with SBF through interfacial and cell-mediated reactions (Kaur et al., 2014). This apatite layer mimics the chemical and crystallographic characteristics of bone, which allows it to chemically bond to host bone, thus the apatite-forming ability is a crucial parameter for bone repair. The XRD patterns (Figures 4B,C) showed the crystalline phase change of the scaffolds after soaking in SBF for 3 and 7 days. The sharp diffraction peaks appeared at 26, 32, 39, 46, 49, and 53°(2θ) was the crystal face of (002), (211), (310), (222), (213), and (004) belonged to HA characteristic peaks (Zhao et al., 2016). In addition, the quantitative analysis by EDS (Tables 1, 2) showed that the scaffold without MNBG had little Ca and the Ca/P ratio in PLGA-MNBG scaffolds after mineralization were among 1.1–1.8, which were similar to the theoretical Ca/P ratio (1.67) of hydroxyapatite (Xu et al., 2010), suggesting the formation of HA. The 0% MNBG group did not show any mineralization peaks even at day 7 and the apatite-forming rate was dependent on the concentration of MNBG, as the amount of MNBG increased and the mineralization time extended, there appeared more diffraction peaks and became more remarkable. However, the 20% MNBG and 30% MNBG groups showed more completed diffraction peaks than 10% MNBG and 40% MNBG groups, indicating their better apatite-forming ability, which was in accordance with the degradation rate accelerated with the increase of MNBG. In summary, the incorporation of MNBG dramatically improved the mineralization performance.

**Table 1:** The ratio of Ca to *P* after scaffolds immersion in SBF for 3 days.

	**Ca(%)**	***P* (%)**	**Ca/P**
0% MNBG	2.64	97.1	0.02
10% MNBG	8.1	2.14	3.78
20% MNBG	19.75	10	1.97
30% MNBG	59.62	36.52	1.63
40% MNBG	56.85	31.0	1.83

**Table 2:** The ratio of Ca to *P* after scaffolds immersion in SBF for 7 days.

	**Ca(%)**	***P* (%)**	**Ca/P**
0% MNBG	1.54	98.4	0.01
10% MNBG	10.97	7.08	1.54
20% MNBG	16.41	13	1.26
30% MNBG	31.5	22.77	1.38
40% MNBG	48.51	41.03	1.18

The ion release in scaffolds was detected at day 3 and 7 after soaking in SBF (Figure 4). The results showed that 0% MNBG group had little changes in ion concentration, 10% MNBG group showed the same trend as 0%MNBG, because the less MNBG was better wrapped in PLGA. Scaffolds with 20, 30, and 40% MNBG showed that the Si (Figure 4D) and Ca (Figure 4E) concentration had a sharp raise after 3 days, while the P concentration (Figure 4F) showed a trend of continuous decline. It could be explained that because of the contact between SBF and MNBG in the scaffold, the H^+^ in SBF will be replaced and the soluble Si-OH will be formed on the surface of MNBG (Martínez et al., 2000; Hesaraki et al., 2010). In this process, Ca ions will be largely dissolved from the surface of MNBG, resulting in a sharp increase in the concentration of Ca ions in SBF. In addition, the mutual substitution of H^+^ and Ca^2+^ leaded to the continuous increase of the pH of SBF, thus accelerating the generation of Si-OH and the depolymerization of silica network in MNBG. The P concentration decreased significantly in this process, which was the result of low initial P amount and constant consumption of P during the formation of apatite, while the initial amount of Si and Ca was large and the dissolution rate was higher than the consumption rate of apatite formation, reflecting the increase of concentration as a whole. As the extension of mineralization time, the amount of apatite formed kept increasing, and the consumption of Ca and P also increased, so their concentration in SBF showed a trend of continuous decline.

### *In vitro* Biocompatibility and Osteogenesis

**Figure 5:**
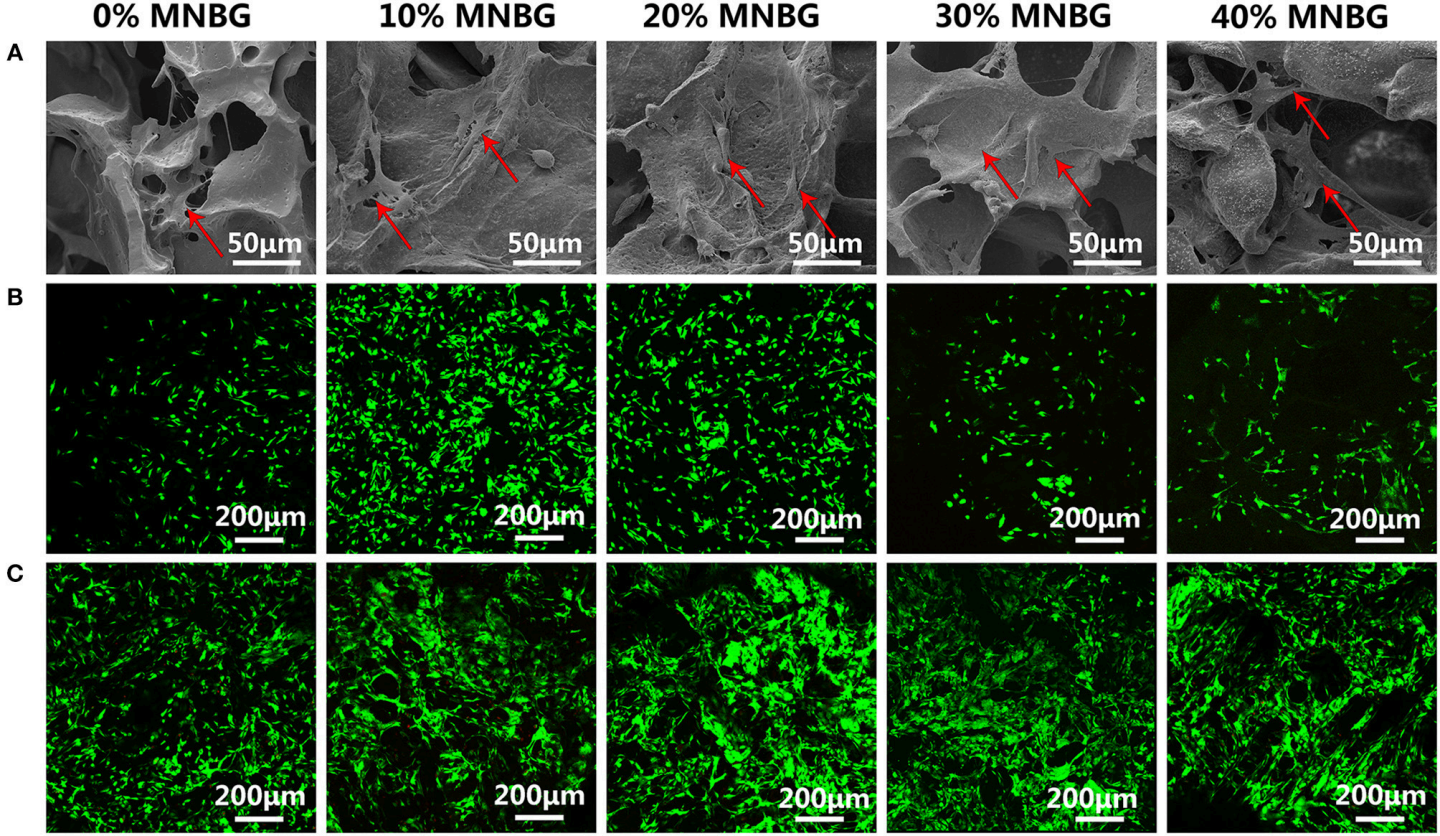
Cell attachment and cell viability evaluation on scaffolds. **(A)** SEM images showing the mBMSCs attachment and spreading at day 1 (Red row in SEM images). **(B,C)** Cell viability detected by Live-Dead assay suggesting the good cell viability on the surface of scaffolds at **(B)** day 1 and **(C)** day 5. Green represents living cells and red represents dead cells.

**Figure 6:**
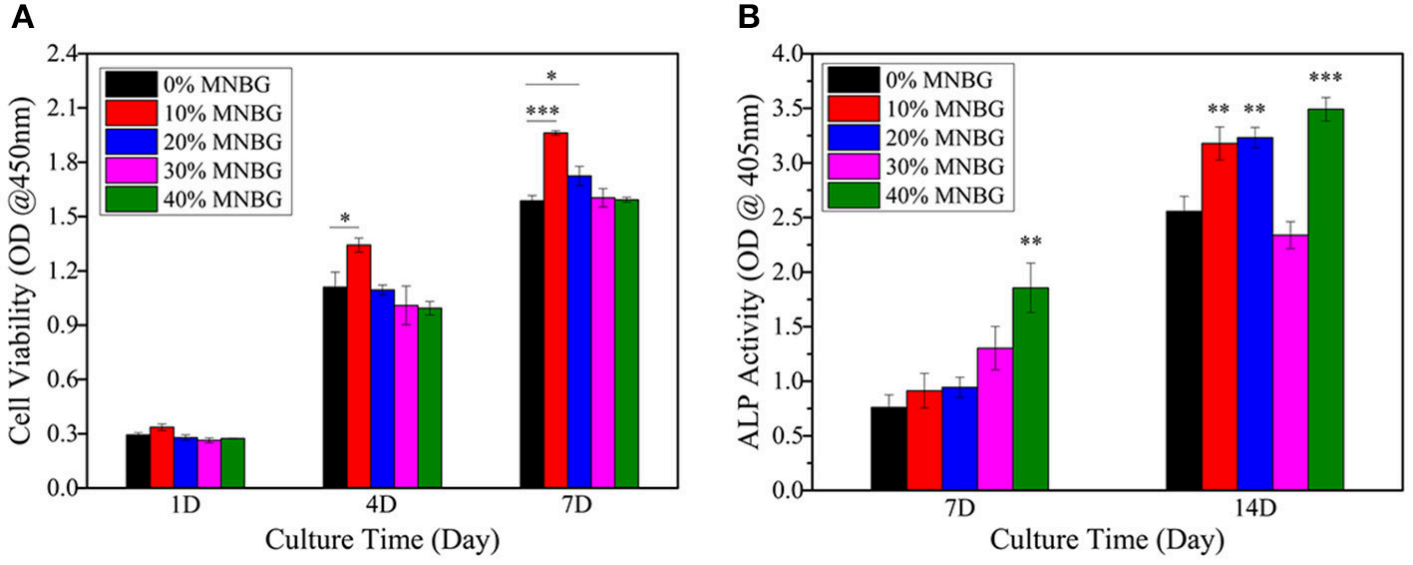
Proliferation and ALP activity analysis of mBMSCs on PLGA-MNBG scaffolds. **(A)** CCK-8 assay indicating the cell proliferation at day 1, 4 and 7. **(B)** ALP activity test cells after incubated on various scaffolds for 7 and 14 days. (^*^p < 0.05, ^**^p < 0.01, ^***^p < 0.001 compare to 0% MNBG).

Cell attachment and viability on the implants is a crucial factor for promoting osseointegration. Here, BMSCs were chose to be used for investigating the cell biocompatibility of scaffolds, due to their good record in bone regeneration (Yu et al., 2017). The morphology and adhesion properties of mBMSCs cultured on the scaffold at day 3 were detected by SEM (Figure 5A). The SEM images revealed that cells had adhered with well-spread morphology on all groups of scaffolds surface with extended pseudopod (even cross over the pores), and scaffolds with MNBG had larger spread area. Thus, the incorporation of MNBG could promote cell spreading. The mBMSCs viability cultured on the scaffold surface was evaluated by Live-Dead fluorescence staining under CLSM at day 1 and day 5 (Figures 5B,C). The images showed that almost all cells were live (green fluorescence), only few dead cells (red fluorescence), the cells were growing and spreading around the porous structure and the density of live cells were higher with the culture time, indicating good cellular activities of scaffolds. Among the MNBG incorporated groups, 10% MNBG and 20% MNBG groups showed significantly better cell viability than high concentration groups at day 1 as compared to 0% MNBG, because MNBG could release abundant Si^4+^ and Ca^2+^ ions and generate alkaline environment which affected the cell behavior in the early culturing (Zheng et al., 2018). As a whole, the incorporation of MNBG could significantly enhance the mBMSCs attachment on PLGA scaffolds. The proliferation of mBMSCs cultured on the composite scaffold were quantified by CCK-8 assay at day 1, 4, and 7 (Figure 6A). The OD value suggested the cell number and cell activity on the scaffolds. The results showed that cells were significantly increased for various groups as the culture times (Figure 6A). As compared to 0% MNBG (PLGA), the 10% MNBG group significantly improved the cell viability and proliferation at day 4 and day 7.

The earlier marker of osteogenic differentiation is signified by ALP activity and it plays an important role in evaluating osteogenesis effects *in vitro* and *in vivo* for implants (Thakur et al., 2016). The ALP activity of mBMSCs on the scaffold was quantified at day 7 and 14 (Figure 6B). The result indicated that the significant improvement of MNBG groups for ALP expression than 0% MNBG group at day 14, which was probably attributed to the Si, Ca ions release from the MNBG through activating the MAPK signal path (Mao et al., 2017). However, 30% MNBG group showed a very low (lower than 0% MNBG group) ALP activity at day 14, which may be due to the excess MNBG in scaffold inducing an alkaline micro-environment which could interference mBMSCs differentiation and decrease the ALP expression (Shen et al., 2012; Siqueira et al., 2017). The 40% MNBG group still own higher ALP activity was probably because of the lots of loss from the scaffolds which resulted in the decreased MNBG concentration. As a word, the low concentration of MNBG in scaffolds could significantly promote ALP expression of mBMSCs.

### *In vitro* Biocompatibility and Angiogenesis Studies of HUVECs

**Figure 7:**
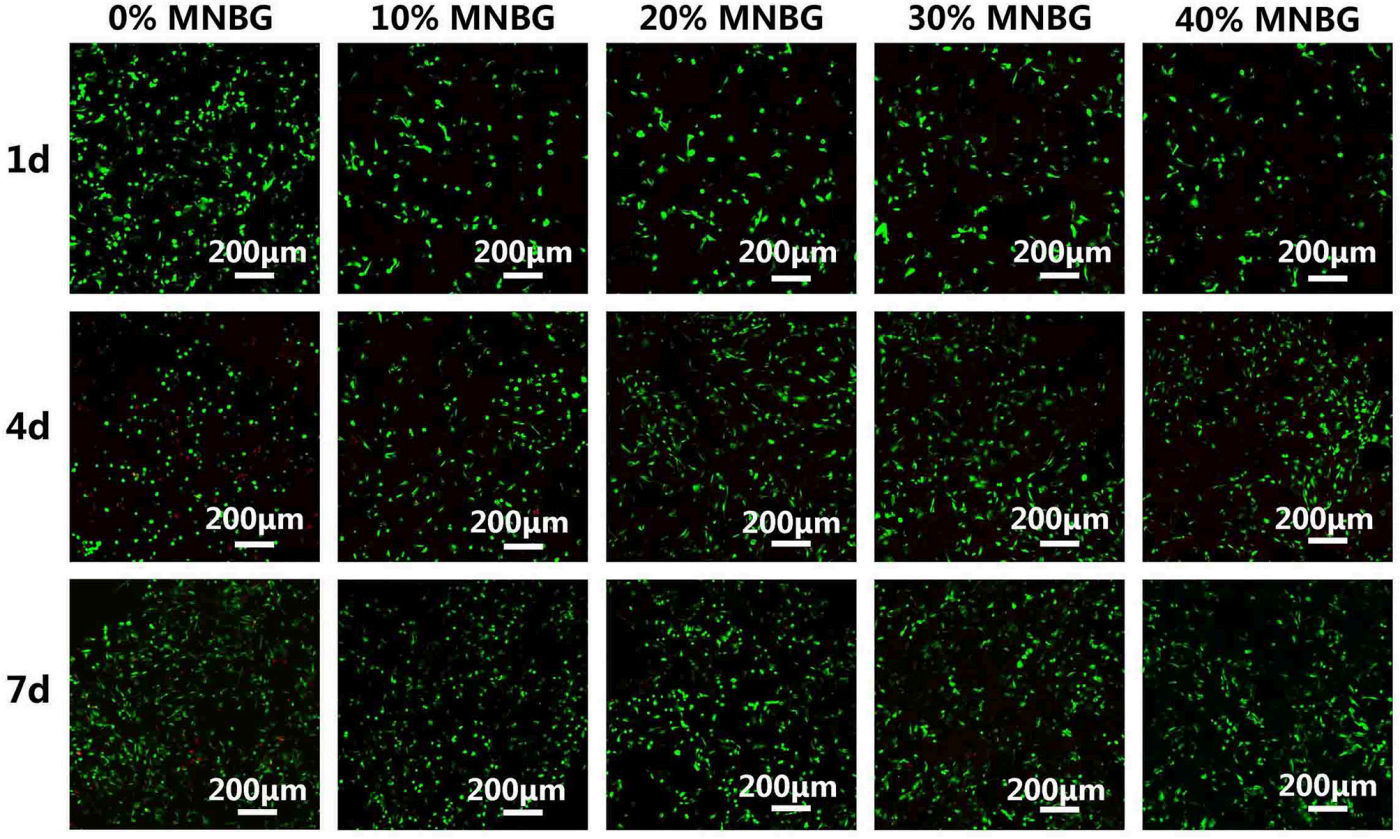
HUVECs viability evaluation cultured on PLGA-MNBG scaffolds with various MNBG concentrations detected by Live-Dead assay at day 1, 4, and 7. Green represents living cells and red represents dead cells.

The angiogenesis capacity of biomaterials was rather important for enhancing their bone regeneration applications. Here, we investigated the effect of PLGA-MNBG scaffolds on the angiogenesis of HUVECs. HUVECs viability cultured on the scaffold surface was evaluated by Live-Dead fluorescence staining under CLSM at day 1, 4, and 7 (Figure 7). The images showed that the cells lived well on the scaffold surface (green fluorescence), only few dead cells (red fluorescence) on the scaffold was observed with MNBG incorporated. The density of live cells was higher with the culture time. For pure PLGA scaffold, though it had cells proliferation on its surface, there were also much more dead cells compared to composite scaffold with MNBG, this might be due to the acid environment resulted from PLGA degradation (Kido et al., 2017). As a whole, HUVECs could survive well on the composite scaffold with MNBG, it revealed that the MNBG had the potential to promote cell survival and angiogenesis (Dashnyam et al., 2017).

**Figure 8:**
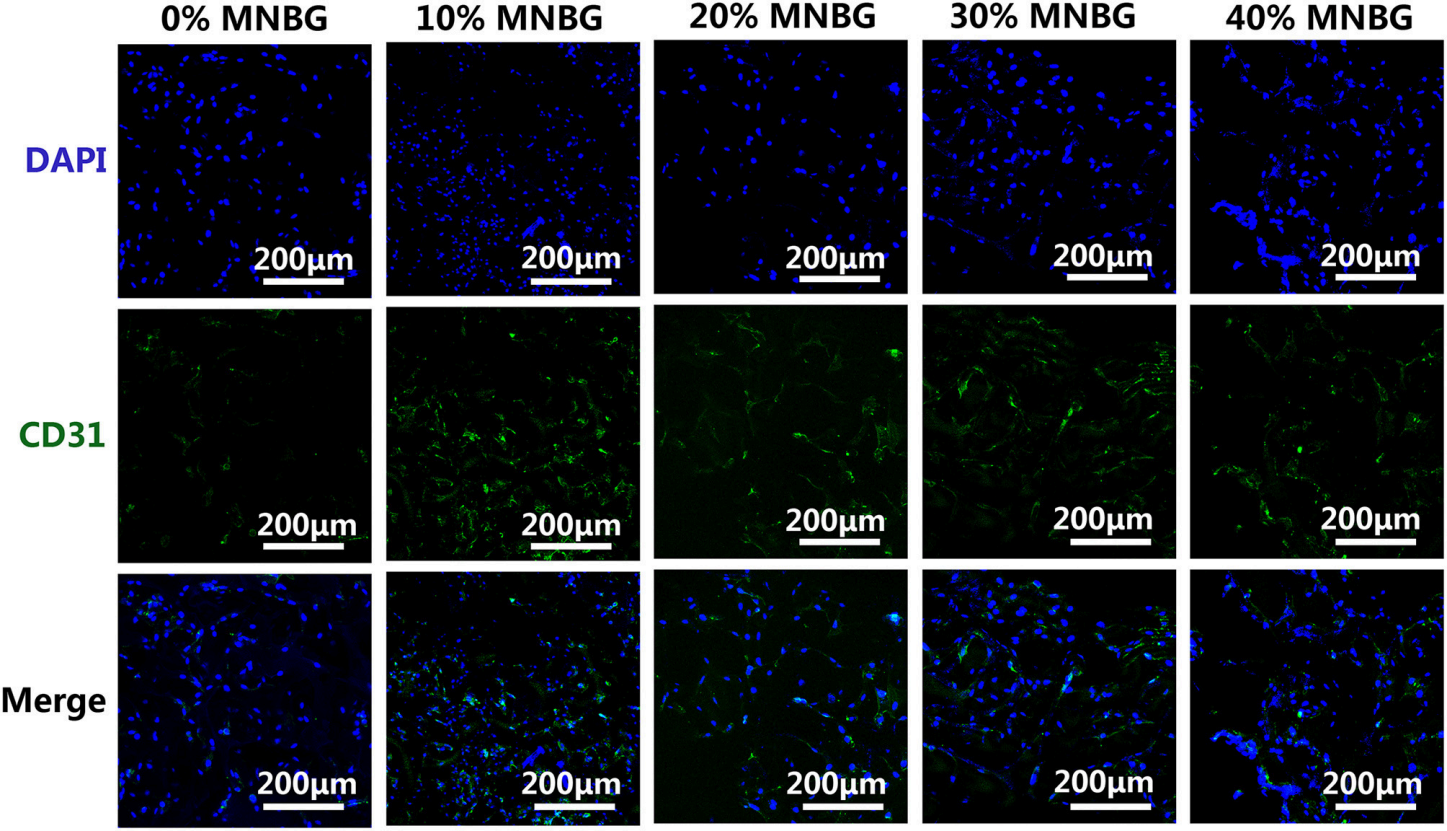
Expression of CD31 in HUVECs by an immunofluorescence assay. Immunostaining images of CD31 (green) and DAPI (blue) for different scaffolds.

The further *in vitro* angiogenesis of HUVECs affected by scaffolds was performed through investigating the protein expression by immunofluorescence staining of CD31 (transmembrane protein) which is expressed in early vascular development (Gao et al., 2017; Zhao et al., 2018b). The immunofluorescence assay image at day 3 (Figure 8) showed that a large amount of CD31 positive staining in scaffold with MNBG was observed (green), little could be found in 0% MNBG group, suggesting that the MNBG groups expressed higher levels of CD31. These results indicated the MNBG could significantly promote the expression of angiogenesis marker (CD31) and might have meaningful effect in bone regeneration.

## Conclusions

In summary, we fabricated the porous PLGA-MNBG nanocomposite scaffolds with excellent *in vitro* osteogenesis and angiogenesis performance through a simple phase separation method. The mechanical property and pore diameter of PLGA-MNBG scaffold was significantly improved due to the incorporation of MNBG particles. In addition, the *in vitro* cell experiments demonstrated that PLGA-MNBG scaffolds significantly enhanced the mBMSCs attachment, proliferation, and osteogenic differentiation at a low MNBG concentration. Furthermore, the functional MNBG particles also significantly enhanced the *in vitro* angiogenesis (CD 31 expression) for HUVECs. This study shows that PLGA-MNBG is a potential promising porous scaffolds with high bioactivity for enhanced bone repair and regeneration.

## Author Contributions

TT wrote the manuscript. WX, WG, and GW collected the literature. LZ and GM provided general idea. BL, ZL, and XC edited the manuscript. All authors read and approved the final manuscript.

### Conflict of Interest Statement

The authors declare that the research was conducted in the absence of any commercial or financial relationships that could be construed as a potential conflict of interest.
