# Corrigendum: Micro-Nano Bioactive Glass Particles Incorporated Porous Scaffold for Promoting Osteogenesis and Angiogenesis *in vitro*

**DOI:** 10.3389/fchem.2022.891108

**Published:** 2022-04-26

**Authors:** Ting Tian, Weihan Xie, Wendong Gao, Gang Wang, Lei Zeng, Guohou Miao, Bo Lei, Zhanyi Lin, Xiaofeng Chen

**Affiliations:** ^1^ Guangzhou Higher Education Mega Center, School of Medicine, South China University of Technology, Guangzhou, China; ^2^ Department of Biomedical Engineering, School of Materials Science and Engineering, South China University of Technology, Guangzhou, China; ^3^ National Engineering Research Center for Tissue Restoration and Reconstruction, Guangzhou, China; ^4^ Key Laboratory of Biomedical Materials and Engineering, South China University of Technology, Ministry of Education, Guangzhou, China; ^5^ Key Laboratory of Oral Medicine, Guangzhou Institute of Oral Disease, Stomatology Hospital of Guangzhou Medical University, Guangzhou, China; ^6^ Instrument Analysis Center, Frontier Institute of Science and Technology, Xi’an Jiaotong University, Xi’an, China; ^7^ Department of Cardiology, Guangdong General Hospital, School of Medicine, South China University of Technology, Guangdong, China

**Keywords:** bioactive glass, micro-nano particles, nanocomposites scaffolds, bone regeneration, osteogenesis

In the original article, there were errors. In the section ***In Vitro* Cellular Evaluation of Composite Scaffold**, “*Cell Culture*”, page 3, an acronym was not given in full at the first mention. The corrected sentence is as follows:

“The scaffold (2 mm height and 8 mm diameter) were placed into the 48-well plates, sterilized by immersing in 75% ethanol overnight and washed with phosphate-buffered saline (PBS) for three times by 30 min interval.”

Consequently, in the next section “*Cell attachment*”, the first sentence is corrected as follows:

“For cell attachment testing, the scaffolds were harvested at 3 days and washed with PBS for twice, fixed with 2.5% glutaraldehyde at 4°C for 4 h”

In **Conclusions**, page 9, there was a typo in which “was” was used instead of “were,” The corrected sentence is as follows:

“The mechanical property and pore diameter of PLGA-MNBG scaffold were significantly improved due to the incorporation of MNBG particles.”

In the next sentence, the word “cell” was used incorrectly in “the *in vitro* cell experiments.” The corrected sentence is as follows:

“In addition, the *in vitro* experiments demonstrated that PLGA-MNBG scaffolds significantly enhanced the mBMSCs attachment, proliferation and osteogenic differentiation at a low MNBG concentration.”

Figure 4, page 6, was incomplete. The corrected figure is below.

**FIGURE 4:**
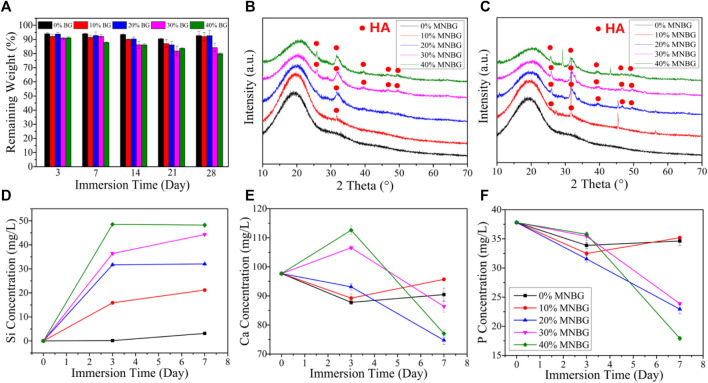
Biodegradation and apatite-forming ability of composite scaffolds in SBF. **(A)** Mass loss behaviors of scaffolds in SBF during 28 days immersing. **(B,C)** XRD patterns of scaffolds after soaking in SBF for 3 days **(B)** and 7 days **(C)**. **(D–F)** Ions release curves of scaffolds for **(D)** Si; **(E)** Ca; **(F)** P after soaking in SBF for 3 and 7 days.

In Figure 5C, the fluorescent image on sample 40% MNBG was provided incorrectly. The corrected figure is above.

**FIGURE 5:**
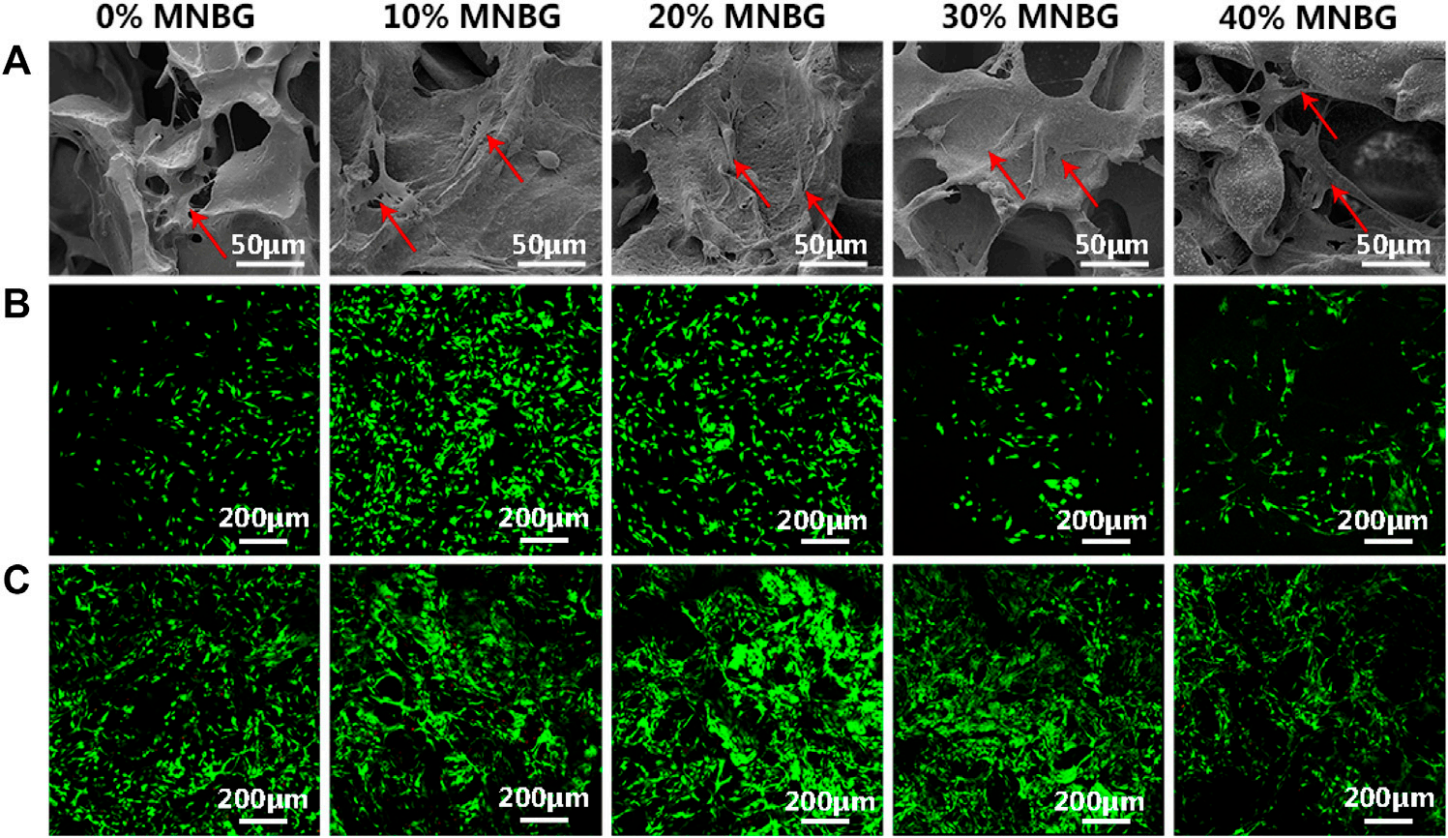
Cell attachment and cell viability evaluation on scaffolds. **(A)** SEM images showing the mBMSCs attachment and spreading at day 1 (Red row in SEM images). **(B,C)** Cell viability detected by Live-Dead assay suggesting the good cell viability on the surface of scaffolds at **(B)** day 1 and **(C)** day 5. Green represents living cells and red represents dead cells.

The authors apologize for these errors and state that this does not change the scientific conclusions of the article in any way. The original article has been updated.

