# Supplementary material for: Micro-Nano Bioactive Glass Particles Incorporated Porous Scaffold for Promoting Osteogenesis and Angiogenesis in vitro
Source: Front Chem. 2019 Mar 29;7:186. doi: 10.3389/fchem.2019.00186 (PMC6449679; doi:10.3389/fchem.2019.00186)
Supplement: Supplementary file 1 [file Presentation_1.pdf]

Supplementary Material

Micro-Nano Bioactive Glass Particles Incorporated Porous Scaffold for Promoting Osteogenesis and Angiogenesis *in vitro*

Ting Tian [a, c, d], Weihan Xie [b, c, d], Wendong Gao [b, c, d], Gang Wang [b, c, d], Lei Zeng [b, c, d], Guohou Miao [g], Bo Lei [f] *, Zhanyi Lin [a, e]*, Xiaofeng Chen [b, c, d]*

*** Correspondence:**

Prof. Bo Lei, E-mail: rayboo@xjtu.edu.cn

Prof. Zhanyi Lin, E-mail: linzhanyi@hotmail.com

Prof. Xiaofeng Chen, E-mail: chenxf@scut.edu.cn

1 Supplementary Figure

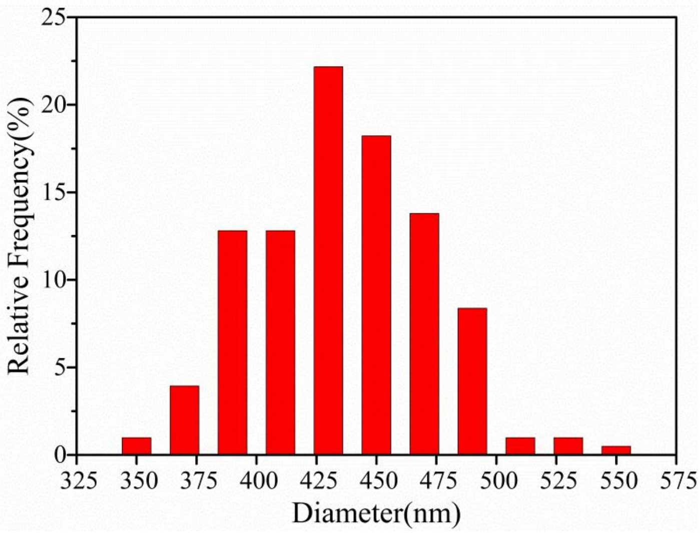

Fig. S1 Particles diameter distribution of micro-nano bioactive glass (MNBG).

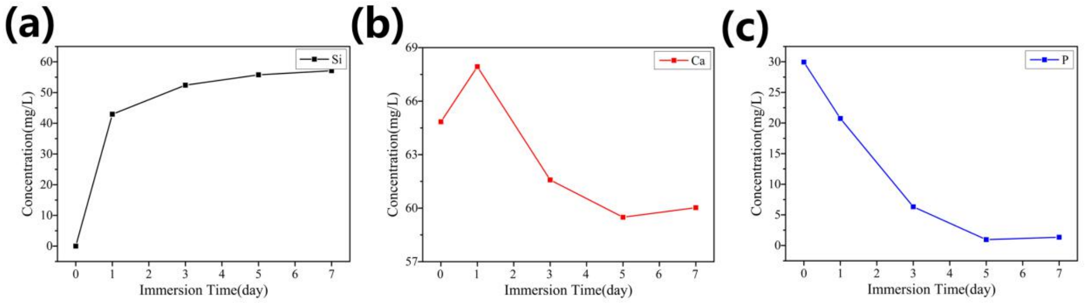

Fig. S2 Bioactive ions release curves of MNBG for (a) Si; (b) Ca; (c) P.

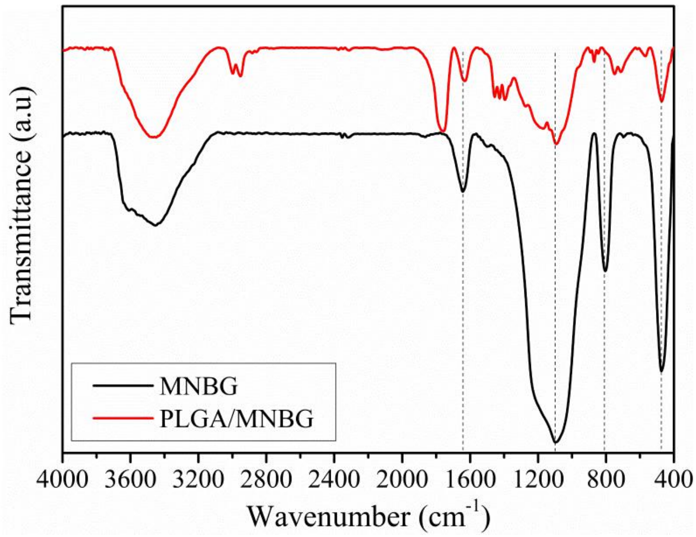

Fig. S3 FTIR spectra of MNBG and PLGA-MNBG.

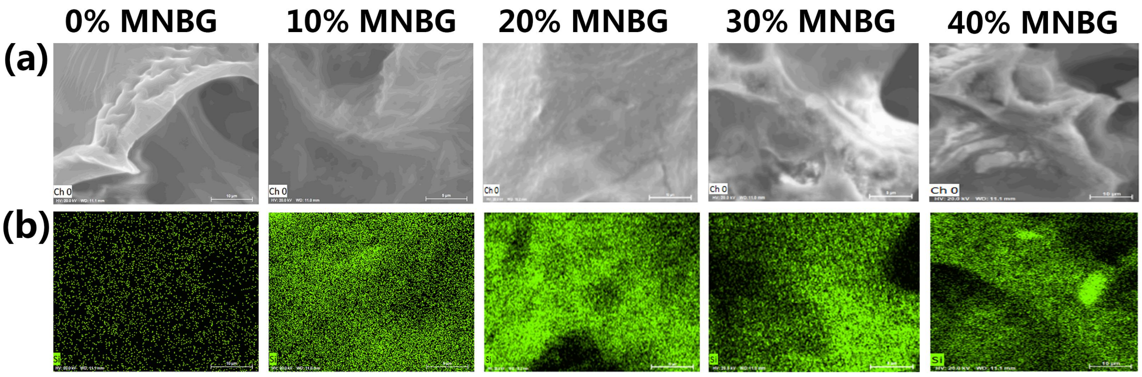

Fig. S4 EDS mapping of PLGA-MNBG scaffolds.(a)The SEM image of the scaffold and (b)Si distribution of the MNBG in the scaffold.
